# New Frontiers in the Production of Functional Recombinant Proteins

**DOI:** 10.3390/bioengineering12040351

**Published:** 2025-03-28

**Authors:** Xin Luo, Yi Ma, Qi Xiang

**Affiliations:** 1 Guangdong Provincial Key Laboratory of Bioengineering Medicine, Institute of Biomedicine, Jinan University, Guangzhou 510632, China; luoxin@stu2023.jnu.edu.cn; 2 Biopharmaceutical R&D Center of Jinan University Co., Ltd., Guangzhou 510632, China; 3 School of Biology and Biological Engineering, South China University of Technology, Guangzhou 510006, China

Recombinant protein technology, a pivotal subset of biotechnology, facilitates the generation of specific proteins via recombinant DNA techniques, predominantly within heterologous expression systems. This field has witnessed remarkable progress, particularly within the realms of medical science, biopharmaceuticals, agriculture, and biotechnological applications [1].

The initial step involves the insertion of the target protein’s gene into an expression vector, such as a plasmid or viral vector [2]. This Special Issue compiles 10 groundbreaking papers in the domain of active peptide and protein biosynthesis, as well as their applications and efficient expression systems, which have substantially contributed to our understanding of cutting-edge technologies in bioactive protein research.

The rapid advancement of modern biotechnology has enabled the production of recombinant proteins with tailored functionalities. This progress is largely attributed to innovations in protein engineering, which allow for the optimization of protein structures to meet specific functional requirements. Techniques such as directed evolution and rational design have been employed to enhance the properties of proteins, making them suitable for various applications in the biomedical and industrial sectors [3]. Moreover, recombinant proteins have played a crucial role in overcoming limitations of traditional drug delivery systems, with many proving highly efficient and some receiving FDA approval [4]. Despite certain advancements, the yield and practical applications of recombinant proteins remain suboptimal.

The production of recombinant proteins in microbial systems has revolutionized the field of biochemistry. However, downstream challenges include the formation of inclusion bodies (IBs) [5], protein inactivity [6], and, in some cases, the complete absence of protein production [7]. Two articles in this Special Issue present effective methodologies for the efficient expression and application of recombinant proteins from diverse perspectives.

Selecting the appropriate host cells for protein expression is crucial, followed by the optimization of expression systems. Yi Ma [8] utilized *E. coli* BL21 (DE3) as host bacteria and recombinantly expressed the lysis protein ID52-E of *E. coli* phage ID52 for the first time in order to study the application in producing bacterial ghosts (BGs) and to screen for the most efficient preparation system of BGs. λRed homologous recombination technique was used to construct EcNΔaraBAD:: FRT and STΔaraBAD::FRT engineered strains. The characterization involved detecting the lysis curve, inactivated efficiency, cytoplasmic contents leakage, BGs formation efficiency, scanning electron microscopy (SEM), transmission electron microscopy (TEM), and protein expression levels verified by Western blot. The results showed that the lysis effect of ID52-E was significantly better than that of φX174-E, and the arabinose inducible promoter significantly improved the lysis effect of ID52-E. High-quality empty shell BGs with complete structure and lysis pores were observed by SEM and TEM. In summary, the study enhanced the efficient preparation and yield of BGs, establishing a technical foundation for their large-scale production and application.

The second article, by Wenjie Xie et al. [9] focused on the design and expression of a functional fragment of human type I collagen (rhLCOL-I) in *Escherichia coli* (*E. coli*) utilizing a temperature-induced expression system. rhLCOL-I demonstrated exceptional thermal stability and the ability to enhance skin cell migration and adhesion, making it suitable for medical applications. The study establishes a high-yield, cost-effective method for producing collagen fragments suitable for medical applications, demonstrating significant potential for large-scale production.

Recombinant proteins have also been utilized in the field of regenerative medicine, particularly in the treatment of chronic refractory wounds. This Special Issue presents two protocols detailing the application of recombinant proteins in the management of diabetic wounds and aphthous ulcers. Cai Xiang et al. [10] reported the development of a recombinant fibronectin, designated rhFN3C, derived from native human fibronectin. The peptides were cloned into the pET-20b vector and expressed in *Escherichia coli* BL21. These peptides correspond to the amino acid sequences located at positions 1444–1545 (FNIII10) and 1632–1901 (FNIII12–14) in native human fibronectin. Furthermore, in vivo administration of rhFN3C led to a significant reduction in ulcer area and enhanced healing rates in Sprague Dawley (SD) rats, with nearly 80% of the ulcers exhibiting healing by Day 7 in the highest concentration group. This study aimed to develop and assess the efficacy of a thermally stable rhFN3C for its potential role in accelerating the healing of aphthous ulcers (AUs).

Recombinant proteins are essential for studying protein function and structure in laboratory settings. Yunxian Li et al. [11] studied the function of interleukin-33 function. It is known that in diabetic murine models, hyperglycemia suppresses the expression of IL-33 in dermal wounds [12]. Furthermore, the administration of exogenous IL-33 facilitates wound tissue repair by enhancing re-epithelialization and promoting the proliferation of type 2 innate lymphoid cells (ILC2s) [13]. The administration of exogenous recombinant IL-33 has been shown to enhance wound healing in these models by significantly elevating IL-33 levels in wound tissues, augmenting the population of type 2 innate lymphoid cells (ILC2), and facilitating the phenotypic transition of macrophages from the pro-inflammatory M1 to the anti-inflammatory M2 state. This study underscores the potential therapeutic application of recombinant humanized mature IL-33 (rhmatIL-33) in promoting wound healing, particularly within diabetic environments.

Recombinant proteins also demonstrate significant potential for clinical applications such as vaccines, therapeutic proteins (e.g., insulin, growth hormone), and diagnostic reagents. Through recombinant DNA technology, they offer numerous advantages over traditional protein production methods, including enhanced specificity, reduced immunogenicity, and the ability to engineer proteins with tailored properties for specific therapeutic needs. The rapid advancement of modern biotechnology has enabled the production of recombinant proteins with tailored functionalities, while emerging technologies such as plant-derived nanocarriers and homogeneous biosensors further broaden the scope of biotechnological innovation. Six articles in this Special Issue present agriculture, nanoparticles, biomedical, and biotechnological applications of recombinant proteins from diverse perspectives.

In parallel with recombinant protein advancements, novel drug delivery systems are reshaping therapeutic strategies. Ye Wang et al. [14] explored the potential of plant exosome-like nanoparticles (PELNVs) as biological shuttles for transdermal drug delivery. Through systematic analysis of extraction methods (e.g., ultracentrifugation, microfluidics) and physicochemical characterization, the review demonstrated that PELNVs exhibit exceptional deformability, skin penetration efficiency, and biocompatibility. These nanoparticles enhance transdermal delivery of both small molecules and macromolecular drugs while minimizing immunogenicity, offering a cost-effective platform for localized therapies and gene delivery. The integration of PELNVs with recombinant therapeutic proteins (e.g., cytokines or growth factors) could synergistically address challenges in chronic wound management and dermatological applications, complementing traditional recombinant protein delivery systems [15].

The application of biotechnology extends beyond medical fields into agricultural optimization. Zhanyuan Feng et al. [16] conducted a comparative proteomic analysis of diploid and tetraploid watermelon (*Citrullus lanatus* L.), revealing significant morphological and physiological divergences. Tetraploid variants exhibited larger leaves/fruits, enhanced photosynthetic rates, and distinct protein expression profiles, with only 8 of 21 differentially expressed proteins showing correlation with mRNA levels. This work not only advances polyploid breeding strategies but also underscores the potential of recombinant protein technologies in modulating crop traits, for instance, engineering enzymes to enhance nutrient assimilation or stress resilience in polyploid crops.

Diagnostic reagent development has been revolutionized by homogeneous immunoassays. Hee-Jin Jeong [17] comprehensively reviewed Quenchbody (Q-body) technology, a reagent-free biosensor platform leveraging antibody-fluorophore conjugates. Q-bodies exhibit antigen concentration-dependent fluorescence recovery, enabling rapid detection of biomarkers ranging from haptens to whole cells. Structural optimization of IgG-based Q-bodies and molecular evolution approaches could enhance their sensitivity, creating synergies with recombinant protein engineering. For example, recombinant single-chain variable fragments (scFvs) could serve as modular components in next-generation Q-bodies, streamlining diagnostics for diseases monitored through recombinant protein biomarkers.

Based on recombinant protein expression, the production process is simple and flexible, making it an excellent nanocarrier for the delivery of immunogens, proteins, and enzymes both in vitro and in vivo. Dóra Nagy-Fazekas et al. [18] selected the receptor-binding domain (RBD) of the SARS-CoV-2 spike protein and utilized various strains of *Escherichia coli* (such as C43(DE3), Shuffle-T7, and HB2151) for the expression of nanobodies. The periplasmic proteins were isolated using osmotic shock, while the cytoplasmic proteins were extracted through ultrasonication. The target proteins were then purified using Ni-NTA affinity chromatography and size-exclusion chromatography. This study provides an efficient and cost-effective solution for the production of nanobodies and RBDs. Moreover, it is not only applicable to the research on SARS-CoV-2 but also extendable to the development of antibodies against other pathogens, demonstrating broad application potential.

The global scientific community is increasingly focusing on the screening and modification of naturally derived antitumor peptides through genetic engineering techniques. Fu Li et al. [19] have made significant progress in this field by screening degradation fragments of a shark-derived peptide, SAIF (Shark Angiogenesis Inhibition Factor). They identified a more stable peptide sequence and further enhanced its stability through cyclization, resulting in a peptide with an extended half-life and robust antitumor activity. In a nude mouse model, ctSAIF did not induce significant changes in body weight or other adverse effects. As a promising antitumor drug candidate, ctSAIF provides a crucial foundation for subsequent drug development and clinical applications.

In the realm of enzymatic engineering, Thi Ngoc Anh Tran et al. [20] highlighted β-glucosidase’s role in converting ginsenosides—bioactive compounds in *Panax ginseng*. The review emphasized recombinant β-glucosidases for efficient deglycosylation, transforming major ginsenosides (e.g., Rb1) into minor forms (e.g., CK) with higher pharmacological activity. This aligns with recombinant protein trends in industrial biocatalysis, where enzyme optimization through directed evolution [3] could maximize conversion yields, bridging biomanufacturing and natural product valorization.

Recombinant proteins are advancing swiftly within the field of biomedicine, offering innovative solutions across diverse applications, including tissue engineering, regenerative medicine, and pharmaceutical sciences. From tailored therapeutics to precision agriculture, the convergence of nanocarriers, biosensors, and enzymatic platforms with traditional expression systems underscores a future where multidisciplinary approaches address yield limitations, functional ambiguities, and translational complexities. The research articles featured in this special edition showcase considerable promise by presenting efficacious research methodologies designed to confront the challenges of suboptimal recombinant protein expression, the equivocality of functional validation, and the intricacies inherent in the practical application of these proteins.
